# Social media use by physicians: a qualitative study of the new frontier of medicine

**DOI:** 10.1186/s12911-016-0327-y

**Published:** 2016-07-15

**Authors:** Lauren Campbell, Yolanda Evans, Megan Pumper, Megan A. Moreno

**Affiliations:** University of Washington School of Public Health, Seattle, USA; University of Washington School of Medicine, Seattle, WA USA; Seattle Children’s Research Institute, Seattle, WA USA

## Abstract

**Background:**

A growing number of physicians are using social media as a professional platform for health communication. The purpose of this study was to understand perspectives and experiences of these “early adopter” physician bloggers and social media users.

**Methods:**

This was an exploratory qualitative study involving in-depth semi-structured telephone interviews of physicians who were early adopters, defined as physicians who used social media to distribute health information. Participants were recruited through snowball sampling. Interview transcripts were manually analyzed for common themes by three separate investigators who came to common conclusions via the constant comparative method.

**Results:**

Seventeen physicians participated in this study, including 35 % females, 76 % pediatricians and 76 % bloggers. Participants identified multiple perceived benefits and barriers to social media use by physicians; further, four major themes were identified. First, participants often saw themselves as rugged individualists who set their own rules for social media health communications. Second, participants expressed uncertainty about boundaries or strategies for social media use. Third, participants described using social media much like traditional media, as a one-way communication platform, rather than as an interactive forum. Finally, participants expressed disparate views regarding the time involved in participating in social media; some felt that time spent on social media was unproblematic to fit into their day while others felt that it was an impediment to patient care.

**Conclusions:**

Uncertainty remains regarding roles and responsibilities of physicians providing medical content within social media forums and few providers appeared to be using the platform to its full potential. Future studies may inform best practices to optimize social media health communication to benefit patients.

## Background

The internet now provides access to health information ranging from factsheets on how to sooth a colicky baby to videos on how to perform a heart transplant [1, 2]. As of 2014, 87 % of U.S. adults reported using the internet and 72 % of internet users reported looking for health information online [3, 4]. Furthermore, a significant number of people accessing health information online reported that the information they found would “likely” or “very likely” influence their future health care decisions [5]. A 2011 study found that both patients and healthcare professionals are increasingly turning to these online resources, including websites, social media and personal research tools for health information [6].

The use of social media sites has grown exponentially over the last few years, with participation in social media increasing from 8 % of internet users in 2005, to 74 % in 2014 [7, 8]. The Oxford Dictionary defines social media as “websites and applications that enable users to create and share content or to participate in social networking” [9]. Social media provides users the opportunity to generate, share, comment on and receive multimedia content distributed amongst multiple users [10]. Social media platforms include collaborative projects (eg. Wikipedia), social networking sites (eg. Facebook) and web logs (blogs) [10].

As social media use has grown, so has peoples’ interest in using it to find or exchange health information. A 2012 survey found that 26 % of health care consumers use a social networking site for health-related purposes, including 11 % who actively participated by posting comments, queries or information about health or medical issues [11, 12]. A 2013 systematic review identified seven key uses of social media for health communication: 1) provide health information on a range of conditions, 2) provide answers to medical questions, 3) facilitate dialogue patient-to-patient and patient-to-health professional, 4) collect data on patient experiences and opinions, 5) health intervention, health promotion and health education, 6) reduce illness stigma and 7) provide online consultations [10]. Social media is unique in that it allows for two-way communication online between creators and consumers of information. Thus, social media may be an ideal platform for mass health communication as well as the potential for addressing specific queries by individuals [13].

Research to date on social media and health has largely focused on the patient experience. A 2014 study illustrated that patients use social media to locate health information, participate in discussion groups and seek support for struggles with illness [14]. This type of information seeking and sharing happens in a variety of ways including discussion forums, chat rooms, instant messaging and even via online consultations with clinicians [15–17]. In some cases, specific social media platforms facilitate health-related dialogue, such as blogs devoted to specific health conditions such as diabetes, or patient portals dedicated to sharing stories such as PatientsLikeMe [7, 18, 19].

Fewer studies have focused on the physician’s role on social media, though physician-generated health information is growing. In a 2010 study of 921 health-related blogs, 43 % of bloggers were physicians [20]. Physician-bloggers have used social media to share health information, network with colleagues, disseminate research, market their practice and engage in health advocacy [14]. However, few healthcare providers have engaged with their patients online [10] and some are hesitant about the acceptability of interacting with patients in this new venue with unclear boundaries [7]. In one 2008 study which analyzed the content of 271 weblogs written by health professionals, researchers found that weblogs offered the opportunity to share narratives, but also risked revealing confidential patient information or reflecting poorly on the author or profession [21].

While some national medical organizations have published social media guidelines, these guidelines often lack specific behavioral guidance or definitions of professionalism [22–25]. More information is needed to understand best practices to optimize social media’s potential for health communication [26]. Guidelines may be informed by understanding the perspectives and strategies employed by “early adopter” [27] physicians who were pioneers in being among the first wave of physicians to use social media professionally to disseminate health information. The purpose of this study was to explore why and how physicians on social media use these new technology tools. Our study aims were to understand perceived benefits and risks of using social media as a health professional, as well as to understand physician’s experiences, perceptions and views on current and future use of social media in healthcare.

## Methods

### Study design

This was an exploratory qualitative research study designed to gain an understanding of physician views on using social media for communication about health. For the purpose of this study, social media was defined as any website that enables users to create and share content or to participate in social networking. Qualitative methods were used for this study because they are the optimum method to provide data on the needs, beliefs, attitudes and values of key populations [28]. Consistent with qualitative research methods, we did not enter into this study with specific hypotheses. Interviews were selected as the primary method of data collection because this approach encourages open information exchange and targeted follow-up questions. One-on-one telephone interviews between a physician and a researcher were recorded verbatim by the researcher via hand-typed notes. This study was conducted between January 2014 and November 2015.

### Participants

The participants in this study were physicians who had at least one year cumulative experience using social media to distribute health information. As social media use for health promotion by physicians is still a relatively new practice, the participants were considered to be early adopters in this field.

### Data collection

Our study aims were to understand perceived benefits and risks of using social media as a health professional, as well as to understand physician’s experiences, perceptions and views on current and future use of social media in healthcare. Interview questions to meet these objectives were developed by study staff and reviewed by several experts in qualitative research. Questions were further revised on the basis of feedback from participants. Given that the phenomenon of social media use by healthcare providers is relatively new, open-ended questions were used to obtain insights from participants. We asked physicians to describe their experiences with using social media for health information, including benefits and challenges, institution support, and perceptions and experiences of their online audiences. Demographic information included gender, race, years in practice, type of practice, and social media tools used.

### Recruitment

Recruitment took place through snowball sampling: a recruitment technique involving identifying an initial purposeful sample, then asking those participants for further suggestions of potential participants who meet inclusion criteria [29–31]. This technique is particularly effective in studies of early adopters since there are usually few individuals involved: they are usually the best resource for determining who else is involved in the field. While recruitment began with pediatricians, we asked that participants recommend physicians from a variety of specialties in a variety of practices for future interviews, including prominent physician bloggers. Providers were sent a standardized email, blog or Facebook message informing them of the purpose and purview of the study and consent information. Upon receiving a positive reply, a telephone call was scheduled to provide additional information, discuss informed consent and conduct the interview.

### Procedure

Prior to the interview, the researcher and participant discussed the study goals and followed informed consent procedures. Interviews were transcribed verbatim by the interviewer via hand-typed notes. Participants were not provided with incentives for their participation. Interviews generally lasted twenty to forty minutes.

### Availability of data and materials

Data is available in the University of Washington Thesis Library database or upon request of the primary investigator.

### Analysis

Analyses were conducted in two stages. First, a preliminary evaluation of transcripts was completed after ten interviews. In these analyses, preliminary coding and discussion was conducted by two investigators to inform early ideas about themes, to assess repetition of benefits and risks, and to consider thematic saturation. After 17 interviews were completed, investigators reviewed transcripts and noted repetition of benefits and risks and saturation of themes. Thus, a formal coding was conducted at that time using an iterative process [32], transcripts were first read individually by three investigators to identify key codes and themes. A discussion was then held between investigators to discuss codes and themes towards a consensus on coding approaches. Transcripts were then re-assessed and the investigators deliberated until consensus was reached on the common themes and concepts expressed by participants using the constant comparative method [28].

## Results

### Participants

**Fig. 1:**
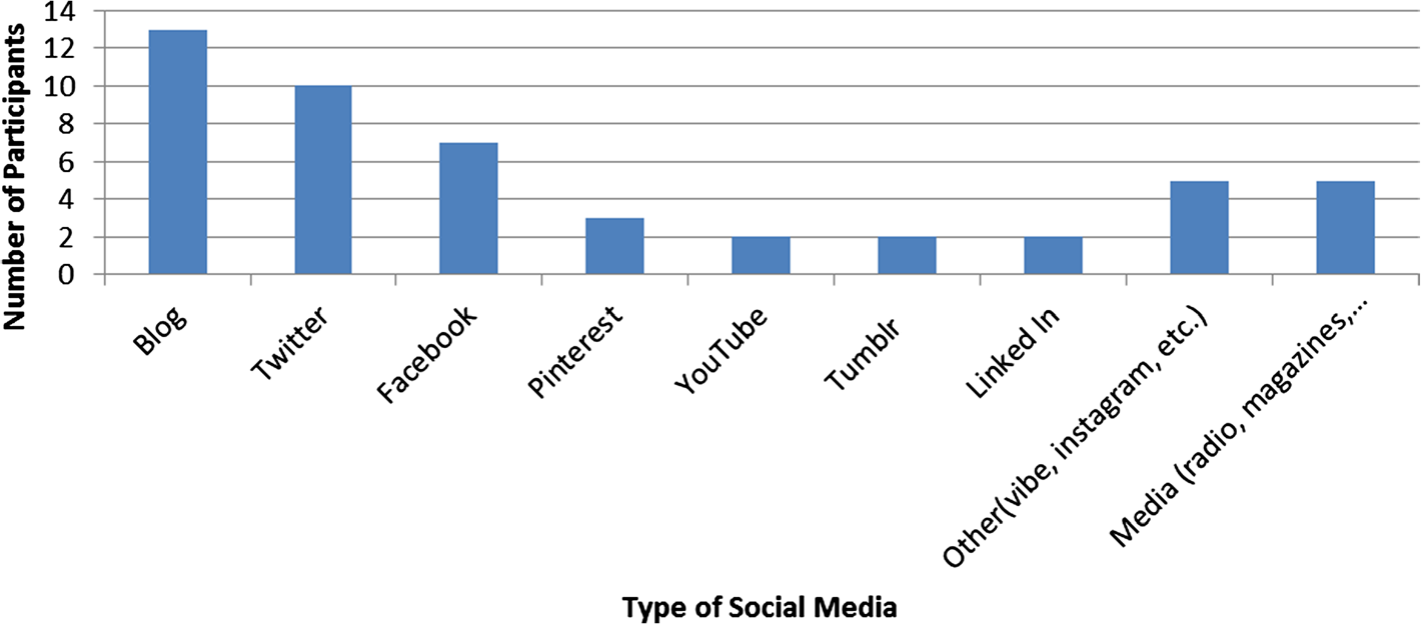
Categories of social media used by participants

**Fig. 2:**
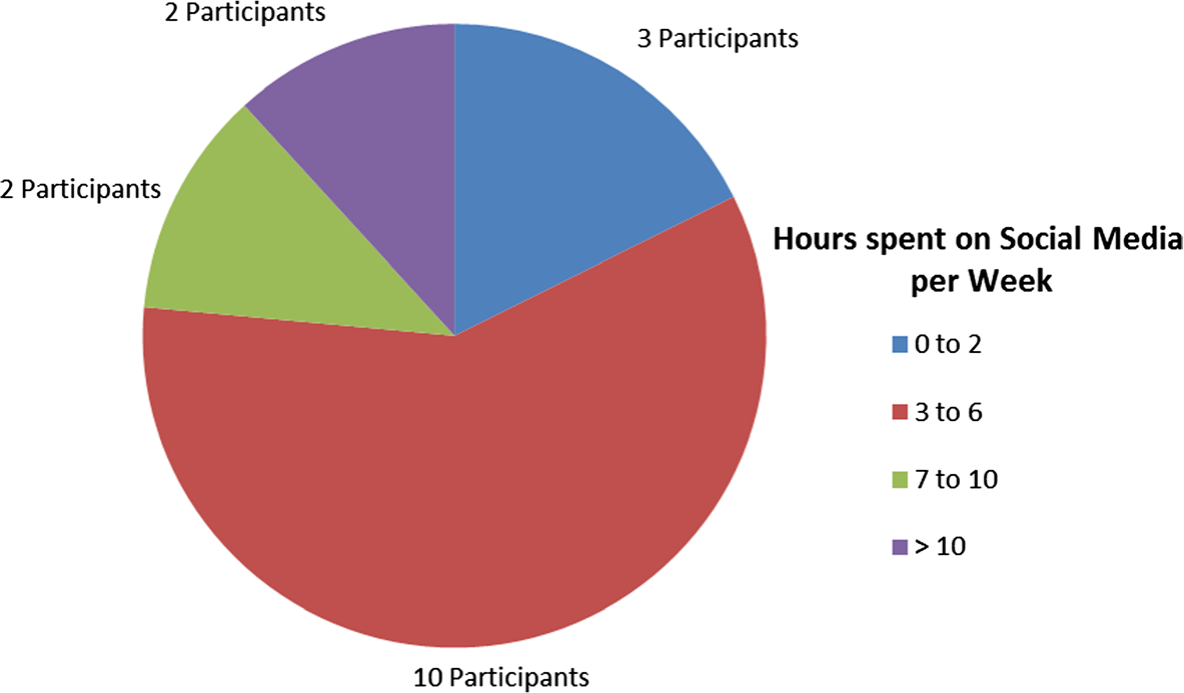
Hours per week spent using social media

Seventeen physicians participated in this study, only one blogger responded and declined participation. Of the seventeen participants, the majority were male (65 %) and Caucasian (82 %) (Table 1). Only one of the participants had practiced medicine for less than five years, with the majority (41 %) having been in practice for eleven to twenty years (Table 1). In terms of specialty, most of the participants were pediatricians (76 %) (Table 1). Almost all of the participants engaged with multiple social media platforms, including Twitter (10), Facebook (7), Pinterest (3), YouTube (2) and Tumblr (2) as well as freestanding Blogs (13) (Fig. 1). Five of the participants also engaged in traditional media such as radio, newspaper columns and magazines (Fig. 1). Although the majority of participants spent three to six hours per week working on social media platforms, a few spent less than two or greater than ten hours per week on social media (Fig. 2). Around one third of participants were compensated for at least some of their work on social media (Table 1).

**Table 1:** Demographics of study participants

Category	Subcategory	N(%)
Gender	Male	11 (65)
	Female	6 (35)
Race	Caucasian	14 (82)
	Asian	2 (12)
	African American	1 (6)
Years in Practice	0–5	1 (6)
	6–10	5 (29)
	11–20	7 (41)
	20–44	4 (24)
Medical Specialty	Pediatrics	13 (76)
	Internal Medicine	2 (12)
	Family Medicine	1 (6)
	Surgery	1 (6)
Compensation for blogging	Yes	5 (31)
	No	11 (69)
	Missing	1

### Benefits and barriers

Findings included a striking variety of perceived benefits and barriers to social media use by physicians. Most physicians voiced the opinion that the benefits of their participation in social media far outweighed any barriers they faced. The perceived benefits of their social media use included forwarding their career or research endeavors, self-improvement through reading others’ tweets and keeping up with the literature, increasing their reach, i.e., their audience, and providing a space for them to openly express their opinions. However, participants also identified several categories of barriers hindering their participation in social media. Examples of these included the time/work requirements, skill requirements, lack of institutional support, fear of saying the wrong thing online and lack of models/guidelines in how to conduct themselves online in their role as physicians.

Physician participants also discussed several benefits and barriers for patients using social media for health information. They perceived that patient benefits may include having increased access to an accurate, trusted and understandable source of health information, that social media is accessible in general and that it provides a way for patients to engage with providers outside the office. Participants also perceived several barriers to patient engagement in social media, including language barriers, poor literacy/education, lack of internet access and worries about the lack of online privacy. In addition, participants identified several mutual benefits for patients and providers. These included increasing communication options and the speed of communication as well as breaking down traditional professional barriers. Finally, social media know-how was described by participants as a mutual barrier to uptake for both physicians and patients. See Table 2 below for a full list of benefits and barriers to social media use by physicians as perceived by participants.

**Table 2:** Participants’ perceived benefits and barriers of using social media

	Benefits	Quotes	Barriers	Quotes
For Providers/ Institutions	Forward Career/Research Marketing, Publicity, Branding Networking/ Sharing ideas New Research/Career Opportunities Low Cost Repository for Information Improvement in Clinic Efficiency	It “doesn’t cost much in terms of money… and it can lead to other opportunities (for the provider). It can lead to more media outreach, speaking engagements, opportunities to teach, promotion for your practice, i.e., ‘free’ publicity.”	Time/Work requirements Keeping current with content Researching content Composing/posting content Listening to/responding to comments Needing to update regularly	“Time is a biggie. And training to some extent, although I think that most people these days get how to use social media. Using it well to communicate health information and thoughtfully so you’re not misunderstood and get in medical or legal trouble, that’s different.”
	Self-improvement Learning (staying current) Listening to patient views/ understanding patient needs Teaching (medical education)	“As soon as the new blood pressure guidelines came out, people started tweeting about them. I know about it ‘cause I’m following people who pay attention to that on twitter. It’s a good way to keep up with what thought leaders are talking about.”	Skill requirements Social media know-how Unfamiliarity with tools Unfamiliarity with etiquette/rules Constantly evolving environment Communication skills Media skills	“When I started, I literally had never heard of a blog before… and so the stumbling block was going from never even using it to being it (social media).” “Number two is that it’s an evolving field.” “Another barrier is knowing how to write. People go to college to learn how to do this. Most doctors don’t know how to do this.”
	Increasing Reach Providing accurate information to more people (correcting misinformation) Amplifier effect (physician ‘voice’) Amplifying ideas Where patients are at, especially kids Where patients get information	“We realized that each of us could see maybe 20 to 25 patients a day, but on social media, we could reach hundreds or thousands of patients a day.” “It seems that everyone turns to social media for information. When I go to google something, I almost always end up on a social media site.”	Lack of institutional support Lack of reimbursement Ignorance of benefits Oldschool mentality Lack of models/guidelines Lack of reimbursement	“People are old in the healthcare system. They’re scared and don’t know how to use the medium…There is a 19^th^ century mentality at the level of academic medicine. I think it’s an old world-new world mentality. Healthcare’s been stuck in this 19^th^ century rut.”
	Independent/Unregulated Venue Place to express opinions It’s FUN Camaraderie	“I found that I really enjoy twitter for learning and connecting with people and building relationships. There aren’t many of us, the physicians in this space. This is the best and easiest place to find them (other physicians).”	Fear of saying the wrong thing Saying something unprofessional Breaching patient privacy (HIPPA) Negative response Providing misinformation	“Barriers like if you post something that you shouldn’t have. You can take back something you’ve said, but to have something out there that can be re-tweeted or re-purposed.”
			Lack of models/guidelines	
For Patients/ Communities	Source of health information Source of ‘accurate’ health information Source of ‘trusted’ health information Source of ‘understandable’ health information Source of ‘current’ health information Source of ‘targeted’ health information Source of information outside the clinic visit	“It can provide accurate information from evidence-based research. I can provide a summary of an article in terms that parents can understand instead of them just reading information from the latest celebrity.”	Poor access Language barriers Mental disability Older generation Lower SES (Medicaid patients) Literacy/Education barriers	“The main reason I don’t use it in my own patient care is because the patients I care for have problems with access. For the most empowered, educated patients, it might be useful. That’s not the kind of people I take care of in an urban underserved hospital.”
	Low cost for patients	The benefit “to the patient is that it’s free healthcare.”	Lack of physicians with similar backgrounds	
	Community Outreach/ Input/ Engagement	“It increases the reach of the message and allows me to interact better. It’s not about broadcasting, it’s about being there and being more accessible for people”	Distinguishing credible information	“the general public not being able to distinguish what’s credible on the internet”
	Accessible	“Just today, I’ve gotten emails, pings, hits, likes from 8 different countries. It runs the gamut. I have people from India or Malaysia who probably make dollars per day to presidents of companies who ask for second opinions. It helps to level the playing field.”		
	Health Behavior Change	“We’re trying to educate people who are misinformed. For good or for bad, social media is very good at changing opinions.”		
Both	It’s fast (quick exchange of information)	“When you email them, you don’t get a response. When you tweet them, it’s literally a one min response.”	Lack of social media know-how	“Honestly, I am my own barrier. I’d never heard the word blog until 5 years ago. I’m still learning. My 14 year-old to tells me how.”
	Improved doctor-patient relationship Breaks down professional barriers Increases communication	“It also allows you to maintain a relationship with your patient population on an ongoing basis. About half your patient population is only going to come in once a year or twice a year. How do you stay in touch with that population, make sure they come back, make sure you can provide them information? It helps them and helps them appreciate you as a physician.”	Lack of privacy	“You can almost trace everybody back. What’s that they say? ‘On social media, it’s written in pen not pencil.”

### Themes

In addition to the benefits and barriers, four major themes were identified: (a) Rugged individualism, (b) Uncertainty, (c) Social media as media and (d) Time constraints. These themes were present across interviews and emerged in response to varied questions and discussions.

#### Theme 1: Rugged individualism

A common sentiment that arose during interviews was that physicians participating in social media perceived themselves as pioneers in the field who would like to make their mark on a new frontier of medicine. Thus, participants stated that they often felt alone or independent in this effort. In the words of one participant, “I think most of us who are doing it (social media) feel a little bit like lone rangers.” Physicians expressed several motivations for their participation. For most, the appeal of social media was described as its ability to serve as an independent platform where they could express their own opinions freely, without administrative interference or oversight. Most participants stated that their participation in social media hinged on the premise that they “have all editorial control” on what is published. Only six of the seventeen physicians interviewed reported any peer review of their publications, where the oversight came from a co-blogger or their own practice. None of the seventeen participants mentioned a formal peer review process. One participant described their experience of clinic-based oversight as follows: “They can choose a question, but the answers are always mine.”

While participants were conscious of their role as physicians online and the responsibility of the information that they promulgated, the majority still described their social media page as a personal platform. One example quote was:

“There’s things I can do personally that I couldn’t as part of my practice or the hospital. I can say what I think is important or not, and laugh and joke. It’s just a nice outlet. I’ve definitely gotten connected with people because of my activities on the canvas. I like the idea that as an individual you can be a thought leader without working with these huge multilevel institutions. The ability to do this as an individual, as a physician, is very cool.”

Even though the study was preferentially targeted towards physicians using social media as a tool to reach patients/communities, participants more often touted its utility for personal branding, marketing and networking than for interacting with patients. One participant described, “if you write your own stuff and other people see it, it’s a good way to self-promote.” Furthermore, several participants highlighted that this drive towards personal promotion resulted in having been granted new research or career opportunities as a result of their engagement with social media, even if this was not their original motivation for joining these platforms.

Finally, most participants described using social media as fun and enjoyable. One participant stated: “It’s actually an incredibly empowering medium as a form of self-expression. If you understand how to use it and communicate with it, then it’s really exciting.” The majority of participants had been involved in traditional media or communications prior to engaging with social media and enjoyed interacting in this new sphere personally as well as professionally.

#### Theme 2: Uncertainty

Participants expressed many levels of uncertainty about their preparedness, their impact, the potential for repercussions and the future of physicians’ presence on social media. Participants described feeling unprepared when they started using social media. Many participants described concerns such as lacking knowledge about how to use certain social media platforms versus others. Others described confusion about the rules or etiquette for use, and how to integrate social media with traditional media. Several participants felt that they were “digital immigrants,” including one physician who had “literally never heard of a blog before starting to write one a year ago.” While all participants articulated that they felt capable of learning through experience, uncertainty remained around practical aspects of social media use; for example, when and how often they should be posting content and responding to comments. In addition, participants struggled to define the line between personal and professional use. While most participants chose to share personal stories of health and family, some wrote solely about medical issues and updates. For example, one participant expressed how they “wanted to story-tell and share information organically, to talk about my experience of raising my children at the same time as giving information on pediatrics and prevention.” While another participant explained how she is “very much being a doctor on social media. I’m not doing it much for personal things.”

Secondly, participants were uncertain about who they reached and the impact they had on their audience. Although most physicians were able to track how many hits they had on their site and where the hits were from geographically, where a “hit” is a click on a page, they could only guess at the demographics of their followers and responders. As one participant responded, “I don’t really know. I assume that I’m reaching a representative sample from our patient population at [our health system], but I don’t know.” It is interesting to note that most participants stated that they guessed they were reaching “white middle-to-upper class English speakers with a high reading level.” In response to a question about who they were reaching through social media, one participant responded that she perceived that her readers were “mostly college educated, higher literacy level because I wasn’t writing it as the third grade reading level. I was writing it in the way I would in college. And the questions I’d get back were written in a similar tone.”

In addition, participants did not have information on the outcomes of their social media content in terms of knowledge, skills or behavior change for their audience. A few physicians noted anecdotally that patients would mention topics that they had posted about online; however, they had no data on behavior change, as described by one participant:

“Right now, our data metrics are flawed. We have [information on] how many hits. We have how many people are following you, etcetera. But we don’t have what happened after they access the information. The only thing we can do is if they actually answer us, ‘These three things I’m gonna change….’ People say I’m gonna change this and change that but we don’t really know.”

One participant pointed out that she perceived a change in the vaccination rate of her clinic population of patients since starting her blog. However, she also speculated that this was more likely to be confounding from new patients coming to her clinic or old patients leaving her clinic based on whether or not they agreed with her views on vaccination posted online than on patients changing their minds.

Thirdly, uncertainty was expressed regarding potential negative repercussions as a result of social media use. Some physicians expressed concern about backlash from the media or an employer based on something another physician had posted, though few felt like they were at risk themselves of making such an egregious error. One participant went as far as to insist this concern was irrational:

“The number one problem is the irrational fear of litigation. I have many different physicians writing for me on different topics. I wanted to get some obstetricians to write about prenatal care and they all said, ‘no.’ Malpractice doesn’t include verbiage about this but they should so people can feel free to write… There’s no precedent for docs being sued for general medical writing.”

Finally, participants expressed uncertainty around the issue of whether or not it is the duty/ obligation of all physicians to provide health information on this new accessible medium. While some participants were insistent that more physicians should be involved, most were equivocal, stating that physicians should only participate if they feel they would enjoy it and are equipped to do so. Physicians in private practice were more likely to view social media as an increasingly important aspect of their practice, as one participant described:

“I think everyone should be using social media in their practices. This is how communication is happening right now. As a pediatric provider, you’re not just communicating in the exam room, but ideally you’re being involved in your community, making your community a better place for children. It really becomes global with the internet.”

Two physicians who reported primarily working in low-resource settings, such as with Medicaid patients, were both less enamored with its prospects of reaching their patient populations, or the idea that every physician should be involved in social media. As one physician expressed, “not every platform is for every person. If you’re interested, there’s a lot of benefit, but if you’re not interested, there’s already a lot of people out there.”

#### Theme 3: Social media as media

A third theme was that physicians tended to describe their use of social media similar to what would be expected in traditional media rather than as an interactive “social” platform. Unlike traditional media in which messages are distributed in a one directional manner, social media allows participants to listen as well as communicate back. However, most physicians in this study described treating this platform as a personal space where they could advertise their ideas or brand their niche in medicine instead of as a bidirectional patient-provider network. As one participant phrased it, “I think it’s a megaphone, like any media. It amplifies. Your voice gets to more people.” Many participants described using social media like a loudspeaker where they could project opinions and post evidence based medicine rather an as a conversational tool, such as this participant’s comment:

“I’m an educator and I use social media to educate and to opine. I like just ranting and raving and telling people what I think about something and causing a raucous. It is the twenty first century Hyde park… you can stand on your soap box and yell and they’ll yell back.”

Another related and important aspect of social media discussed by physicians was the amplification effect. According to participants, the amplification effect is “the ability to broadcast or narrow-cast content. Using different tools, you can amplify a message to a certain population or the population at large.” Several physicians discussed how this amplifying effect was exactly the reason why more physicians need to add their voice to social media, especially in terms of countering vaccine misinformation. Participants viewed it as a powerful tool for reaching patients with important medical information, especially messages concerning public health.

#### Theme 4: Time constraints

Time was a thematic element which seemed to diffuse itself through every part of every interview. Participants adamantly defended their views on whether or not time spent working on social media was a serious impediment versus an easy to fit into their day. For some physicians, the time spent listening to others’ twitter streams and blogs, prepping and composing their posts and responding to comments required that they decrease their patient load or add additional work-time to their already busy week. One participant noted that,

“right now, most of the doctors I know with very successful blogs have quit practicing medicine. They’re now bloggers and not doctors. I see that occurring pretty regularly… I have 12 hour days in the office. It’s very difficult for me to go home and do a lot of blogging.”

Conversely, other participants felt that their time spent on social media could easily fit into downtime at meetings or 15 min before bed and expressed the sentiment that physicians who felt otherwise were mistaken. One participant described:

“I would say, the nice thing about social media is that you can do it in between things. I dedicate thirty minutes a day on average. I bundle all of it so I can reach multiple platforms at once. It’s not a lot of time. That is one of the biggest misconceptions.”

Participants also debated whether using social media for health promotion improved or decreased efficiency in the office. While some physicians postulated that their posts on social media may increase clinic efficiency by decreasing unnecessary patient calls and visits, others believed that it decreased efficiency by distracting providers from direct patient care.

Time also served as a barrier to establishing institutional support for participants’ use of social media. Participants expressed how “institutional backing is lukewarm at best” and how, “apparently, according to our organization, we have other priorities.” Several physicians expressed their frustration with trying to “jump through multiple hoops” in order to partner with their institution or practice. Many of these physicians concluded that the time and effort required establishing a partnership was too great of a barrier and that they would rather continue alone. One participant argued, “I’ve been reluctant and too lazy to deal with the formalities of it. Last thing I want to do is spearhead the activities for my colleagues behind the times.” Time was also discussed in terms of the “21^st^ century conception of time,” where clinical conversations, questions and updates happen immediately, not only in a 15 min office visit. Several participants felt that this was one of the most pressing reasons for physicians to expand into the realm of social media, as described in this quote:

“We have the 15 minutes problem. The amount of time I have to speak with people. I cannot cover everything. Some of my patients I may only see once or twice a year. And in the meantime, I’m learning about things that could benefit them. If they’re following me, they can learn about the things I’m learning about… I think that it expands the visit and the relationship in ways that are really great and really easy.”

However, it also represented a barrier to use, since physicians rarely had the flexibility to respond instantaneously or engage in present-time twitter conversations, as described in this quote:

“Most docs are just so pressed for time as it is, trying to already balance work and life. Finding the time to do something you need to update quite frequently. When there were blogs, to post, you could put out content on your own time. Now that it’s constant with Facebook and Twitter, you really need to update on a more timely bases. It’s almost a living thing that keeps going even while you’re in clinic or at home.”

Overall, time held a conflicted position in the eyes of participants.

## Discussion

In this study of social media use by physicians, we identified multiple perceived benefits and barriers for both physicians who create content and readers who consume this content, as well as several themes. First, participants who engaged in social media often saw themselves as rugged individualists who preferred to set their own rules for social media use. Second, participants expressed significant uncertainty concerning when, how and to what extent they should use social media, and the impact of their use of social media on their followers. Third, participants often reported using social media like traditional media, as a one-way communication platform, rather than as a “social” forum. Finally, participants expressed disparate views regarding the concept of time; some felt that time spent on social media was insignificant and easy to fit into their day while others felt that it was an impediment.

Our first theme was that physicians using social media viewed themselves as “rugged individualists.” This viewpoint is consistent with characteristics of early adopters described in previous literature [33]. Previous studies have found that physicians may be early adopters of other ideas and products, such as pharmaceuticals [34]. As the first generation of physicians using social media, it may not be surprising that these participants value their independence, ability to make autonomous decisions and importance in the field. However, as the next wave of physicians begins to engage in social media, it will be important to temper this independence and enthusiasm with the responsibilities of providing published medical counsel on the internet. This may include instituting peer oversight of published content, demonstrating credibility online in terms of professional licensure, institutional support (from American Academy of Pediatrics (AAP), American Medical Association (AMA), American Academy of Family Physicians (AAFP), etc.), or other certification of informational accuracy and providing more training/mentoring for physicians who choose to engage in social media.

Our second theme described how participants noted uncertainty in their responsibilities around providing a new type of information in a new venue. Key contributing factors included a lack of communication or agreement between the needs of the physicians using social media, the needs of the medical community and the needs of patients. While several medical associations have produced guidelines for social media use among physicians, few have taken into account the goals and needs of physicians who are engaged in social media. Guidelines from the AMA and AAP are narrowly focused on professionalism [25, 35]. The AAFP has developed a more comprehensive guide, with topics ranging from patient privacy to medical board concerns to setting social media policies within a practice [36]. While these guidelines may be helpful for physicians on social media, especially concerning liability, few address physicians’ motivations for using social media and their perceived benefits and barriers to use. These uncertainties represent areas in which physician-social media users could collaborate to develop best practices, similar to the process employed when best practices were developed for email use by physicians [37].

Finally, our third theme illustrated that while our participants were recognized as forerunners in the field, many of them still underutilized the full power of social media tools. Many of our participants described using social media as a “megaphone” or mouthpiece, suggesting they were using it to broadcast their own views. The idea of the loudspeaker may be an artifact of the fact that many of our participants had previous experience in traditional broadcast media, marketing or communications. However, participant responses suggested that they felt unaware of who was listening or what their audience was saying. This key element of social media, the capacity for interactive and two-way communication, appeared to be underutilized. Furthermore, several participants’ comments suggested pessimism about reaching their low income, non-English speaking, low literacy and older patients using social media. Participants voiced that they generally felt that their audiences were most likely middle-upper class Caucasian readers. However, studies on social media have found that African American internet-users are more likely to use social networking sites than their non-Hispanic Caucasian counterparts and that seven of ten households earning less than ten thousand dollars a year are using the internet [13, 38]. Thus, there seems to be a disconnect between who physicians are targeting on social media and who is using social media. As a profession, this is an opportunity to provide greater access to health information for all patients and transmit new knowledge to physicians.

Limitations of this study include the small and purposeful study sample, and the predominance of pediatricians in our sample. We specifically recruited physician-bloggers who were nominated by their peers because of their success, influence and recognition. It was the views of these early adopters that we wanted to capture in this study. However, given that this is a nonrandom sample, our findings cannot be generalized to other populations or the full network of physicians on social media. This study was conducted and analyzed over two years, and social media systems are consistently changing. We did not include longitudinal assessments in this study thus we did not assess whether participants’ usage or attitudes changed over the study period. Furthermore, since our study design was qualitative in nature, causal inference was not the goal. Rather, we wanted to explore major themes and areas of importance for the field in order to guide future research and recommendations.

## Conclusions

Our study is the first to evaluate physicians’ perceptions, goals and challenges in using social media. Our participants described numerous benefits and downsides of using social media, viewed themselves as pioneers in the field facing much uncertainty, and debated whether time spent on social media was feasible. According to our participants and general trends, social media is only going to continue to grow its presence within medical care. Therefore, it will be important to consider how to develop best practices. Study findings may be used by physicians who are considering blogging to understand early adopter perspectives on benefits and risks. A peer review process may benefit physician bloggers by reducing uncertainty and helping physician bloggers connect to others in their field. Further, organizations may wish to develop standards for best practices for physicians on social media. These best practices will benefit from input from physicians, medical organizations and patients. In addition, it will be important for current and future physician-social media users to continue improving their integration of the “social” tools of social media to better connect with their patient- and colleague-followers.
